# Oral microbiome homogeneity across diverse human groups from southern Africa: first results from southwestern Angola and Zimbabwe

**DOI:** 10.1186/s12866-023-02970-2

**Published:** 2023-08-18

**Authors:** Vítor Araújo, Anne-Maria Fehn, Admire Phiri, Jeffrey Wills, Jorge Rocha, Magdalena Gayà-Vidal

**Affiliations:** 1 Centro de Investigação em Biodiversidade e Recursos Genéticos, CIBIO, InBIO Laboratório Associado, Universidade do Porto, Campus de Vairão, Vairão, 4485-661 Portugal; 2 Program in Genomics, Biodiversity and Land Planning, CIBIO, BIOPOLIS, Campus de Vairão, Vairão, 4485-661 Portugal; 3 Departamento de Biologia, Faculdade de Ciências, Universidade do Porto, Porto, 4169-007 Portugal; 4 Department of Linguistics and Language Practice, University of Free State, Bloemfontein, South Africa; 5 University of Zimbabwe, Harare, Zimbabwe

**Keywords:** Oral microbiota, Oral microbiome, Saliva, Exome sequencing, Metagenomics, Socio-economic status, Subsistence methods, African populations

## Abstract

**Background:**

While the human oral microbiome is known to play an important role in systemic health, its average composition and diversity patterns are still poorly understood. To gain better insights into the general composition of the microbiome on a global scale, the characterization of microbiomes from a broad range of populations, including non-industrialized societies, is needed. Here, we used the portion of non-human reads obtained through an expanded exome capture sequencing approach to characterize the saliva microbiomes of 52 individuals from eight ethnolinguistically diverse southern African populations from Angola (Kuvale, Kwepe, Himba, Tjimba, Kwisi, Twa, !Xun) and Zimbabwe (Tshwa), including foragers, food-producers, and peripatetic groups (low-status communities who provide services to their dominant neighbors).

**Results:**

Our results indicate that neither host genetics nor livelihood seem to influence the oral microbiome profile, with *Neisseria*, *Streptococcus*, *Prevotella*, *Rothia*, and *Porphyromonas* being the five most frequent genera in southern African groups, in line with what has been shown for other human populations. However, we found that some Tshwa and Twa individuals display an enrichment of pathogenic genera from the *Enterobacteriaceae* family (i.e. *Enterobacter*, *Citrobacter*, *Salmonella*) of the *Proteobacteria* phylum, probably reflecting deficient sanitation and poor health conditions associated with social marginalization.

**Conclusions:**

Taken together, our results suggest that socio-economic status, rather than ethnolinguistic affiliation or subsistence mode, is a key factor in shaping the salivary microbial profiles of human populations in southern Africa.

**Supplementary Information:**

The online version contains supplementary material available at 10.1186/s12866-023-02970-2.

## Background

With over 700 identified species, the oral microbiome presents one of the largest microbiota of the human body [1–3], and plays an important role in the maintenance of oral and systemic health [4, 5]. Studies of the oral microbiome among populations from diverse geographical and ethnic backgrounds using next-generation sequencing approaches have identified non-culturable bacteria and found that in healthy oral cavities, up to 96% of all bacteria species belong to six main phyla: *Firmicutes*, *Actinobacteria*, *Proteobacteria*, *Fusobacteria*, *Bacteroidetes* and *Spirochaetes* [6]. Perturbations in the ecological balance of the oral microbiome (dysbiosis) have been related with oral diseases like caries and periodontitis, with oral cancer, and with systemic diseases like diabetes, obesity, colon, lung, and pancreatic cancer, human immunodeficiency virus (HIV), autoimmune disease, and systemic inflammation [3, 5, 7–10].

The diversity in environmental conditions and distinct microbial communities presented by different buccal tissues makes it difficult to assess the definition of what constitutes a normal microbiome profile. Saliva with its conglomerate of bacteria provides an easily accessible and non-invasive material for studying the general oral microbiota composition. Several studies have attempted to relate the high diversity of saliva microbiome profiles with distinct diets, lifestyles, environmental conditions, and host genetics [11–21]. Although some of these studies involved diverse human groups relying on different subsistence strategies [11, 12, 16, 20], the available ethno-geographic coverage is still insufficient to obtain a representative picture of the salivary microbiome diversity in human populations, with African groups being particularly underrepresented [22].

While most studies on the salivary microbiome have been based on high-throughput amplicon sequencing of fragments of the hypervariable region of the 16S rRNA gene or on shotgun sequencing, Kidd et al. [23] have shown that the microbiome from saliva samples could also be characterized by using reads that do not align to human DNA sequences obtained with an exome capture approach. This method provides an opportunity to expand microbiome studies to diverse ethnic groups for whom genomic data have been obtained from saliva samples.

The present study makes part of our ongoing research on the genetic diversity of different populations from southern Africa with a particular emphasis on southwestern Angola, where several linguistically and ethnically diverse groups reside in a relatively limited geographic area [24–28]. A key region for understanding human population history, southern Africa has been colonized by three distinct pre-colonial settlement layers. The two most ancient layers are associated with speakers of click languages referred to as “Khoisan”, which belong to three distinct families: Kx’a, Tuu, and Khoe-Kwadi. The first layer is associated with the Kx’a and Tuu languages spoken by the autochthonous peoples of Southern Africa, who traditionally rely on foraging and harbor the highest levels of human genetic diversity in the world [29–31]. The second layer is represented by Khoe-Kwadi speakers descending from eastern African pastoralists, who migrated into the area from East Africa around ~ 2 kya but are presently associated with different subsistence strategies, including pastoralism and foraging [32]. The third layer is constituted by Bantu-speaking farmers migrating from West-Central Africa who reached the area around ~ 1.5 kya [33, 34].

Here, we used the saliva-derived non-human reads generated by exome capture sequencing to characterize the microbial communities of 52 individuals from eight ethnolinguistically diverse populations residing in Angola (Kuvale, Kwepe, Himba, Tjimba, Kwisi, Twa, !Xun) and Zimbabwe (Tshwa) [27, unpublished data] in order to obtain a more accurate picture of the oral microbiome diversity in an understudied region of Africa.

We found homogenous microbiome profiles across the studied populations, except for individuals belonging to the Tshwa and Twa groups, who presented considerably elevated frequencies of pathogenic bacteria belonging to the *Enterobacteriaceae* family. Since both groups are strongly marginalized, we conclude that low socio-economic and health status – not ethnicity or host genetic background – are the major drivers of saliva microbiome differentiation in the studied area.

## Methods

### Population samples

**Fig. 1:**
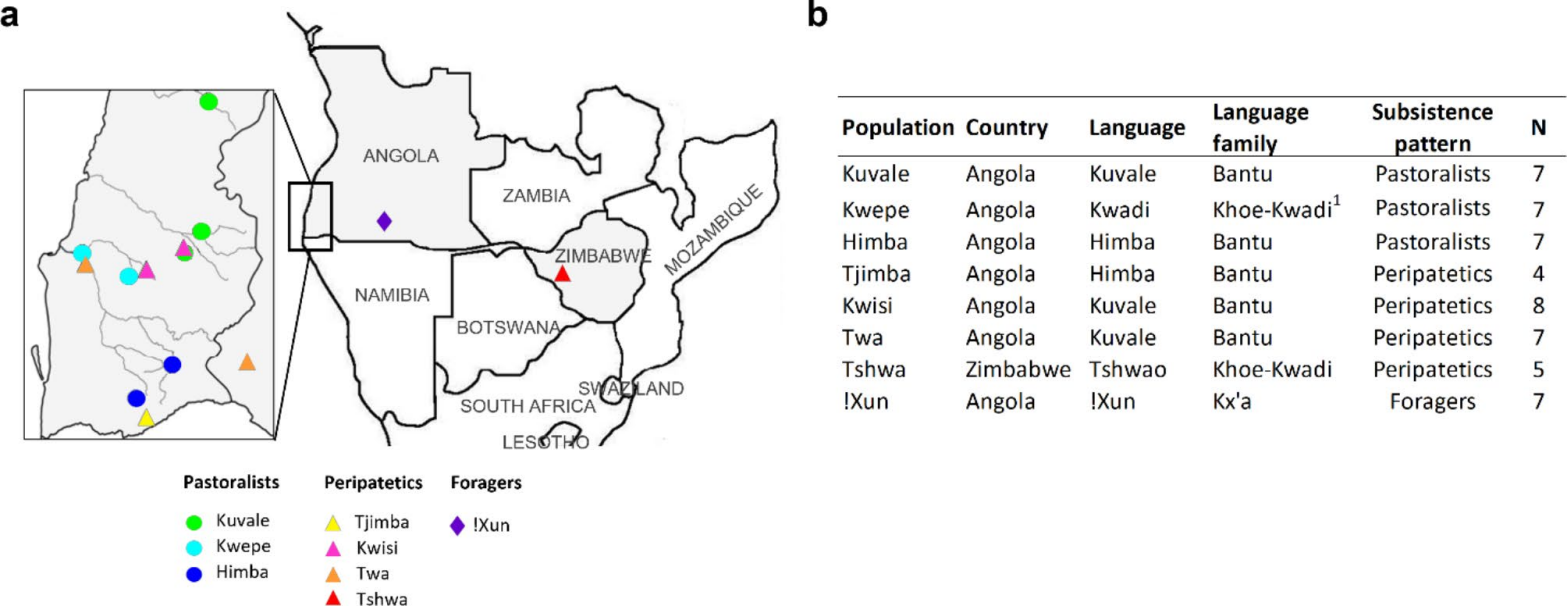
Populations analyzed in this study. **(A)** Map indicating the sampling locations of the studied populations in Southern Africa. Each location is colored by the corresponding population. Country borders are shown in black, the inset shows the Angolan Namib province delimited by a gray contour, and the main intermittent rivers are indicated in light gray. **(B)** Country, language, language family, subsistence pattern, and number of individuals (N) analyzed for each population. Note: while ^1^ Kwadi (Khoe-Kwadi) was the original language of the Kwepe, they presently speak Kuvale (Bantu)

Saliva samples were collected from 52 unrelated individuals (37 males and 15 females) belonging to eight ethnolinguistically diverse populations from Angola (Kuvale, Kwepe, Himba, Tjimba, Kwisi, Twa, !Xun) and Zimbabwe (Tshwa) (see also [24–28]) (Fig. 1; Supplementary Table 1). The data was collected with the written informed consent of all participants and the permission of local authorities, the Provincial Governments of Namibe and Kunene (Angola), and the Ministry of the Local Governance (Zimbabwe). Ethical approval for this study was obtained from CIBIO/InBIO-University of Porto, ISCED, the University of Zimbabwe, and the Tsoro-o-tso San Development Trust boards.

The seven studied groups from southwestern Angola inhabit geographic areas characterized by high linguistic and cultural diversity. The Kuvale, Himba, Tjimba, Kwepe, Twa and Kwisi dwell in the coastal lowlands of the Angolan Namib Desert, which are characterized by an arid and warm climate. As the desert soil is not suitable for agriculture, pastoralism is the sole food production strategy available in the region [35]. The Bantu-speaking Kuvale and Himba cattle herders belong to the Herero pastoral tradition of southwestern Africa, and socially dominate the area. They are surrounded by an array of small-scale populations (Twa, Tjimba, Kwisi) whose livelihoods do not match the traditional division between food-production and foraging and are best described as “peripatetic” [36–38]. While the Tjimba are sometimes considered to be impoverished Himba who lost their cattle, the Twa and Kwisi describe themselves as the autochthonous people of the region and are highly marginalized groups whose origins have often been considered enigmatic [37, 39]. Finally, the formerly Kwadi-speaking Kwepe are small stock herders who may be linked to the early pastoral migration from eastern into southern Africa associated with the Khoe-Kwadi language family [40, 41]. In addition to the Namib populations, we analyzed the Kx’a-speaking !Xun foragers from the neighboring Kunene Province from Angola. This area is characterized by open savanna woodland and makes part of the Kalahari sands landscape unit, with higher rainfall and temperature variance than in the coastal plain [35]. To supplement our Angolan samples with data from other regions of southern Africa, we further included the Khoe-Kwadi-speaking Tshwa from the Tsholotsho District of western Zimbabwe. While their traditional subsistence relied on foraging, they had to leave their traditional hunting grounds in Hwange National Park during the early 20^th^ century and have since experienced considerable levels of social marginalization [42].

### Saliva collection, DNA extraction, library preparation, and sequencing

Details about sample collection and DNA extraction for the Angolan samples are provided in Pinto et al. [24] and Oliveira et al. [25]. The Zimbabwean Tshwa samples were collected in 2015 from the Tsholotsho District. Volunteers were asked to spit up to 2 mL of saliva into tubes containing 2 mL of lysis buffer, which were stored at room temperature until processing. DNA extraction was performed using the Easyspin Genomic DNA Tissue Kit SPDT250 from Citomed according to the manufacturer’s instructions.

Library preparation and expanded exome enrichment were performed using Nextera® Rapid Capture Enrichment kit by Illumina following the protocol version #15037436 v01. DNA concentration for each sample was measured using Qubit 2.0 Fluorometer (Life Technologies) and normalized to 5 ng/µL. The 52 individuals were sequenced in two sequencing runs on an Illumina’s HiSeq 1500 System (Illumina) using 250 cycles in paired-end mode.

### Sequence processing and alignment

FASTQ files were processed to remove low-quality reads by filtering for a Phred Quality Score of 30 (Q30) with Sickle (v1.33) [43] in pair-end mode. Reads that passed the quality filter were aligned to the human genome hg19 using the *-mem* option of Burrows-Wheeler Aligner (BWA) software (v0.7.15) [44]. From the resulting BAM files, we extracted the non-human reads (unmapped reads) using SAMtools [45] and applied further quality filters in accordance with Kidd et al. [23] with PRINSEQ tool [46]. We removed reads with less than 50 bp, reads with a mean quality score < 25, and reads which were exact duplicates. Since PRINSEQ works with FASTQ files, the BAM files were first converted using BEDtools [47].

The high-quality metagenomic reads were blasted against the microbiome reference genomes from the Human Microbiome Project (HMP) [48] (NCBI BioProject PRJNA28331 [Accessed November 19, 2018]) with the software BLAST+ [49] using the option *blastn*, and the best hit for each read was retained. For the species-level binning, we used the most stringent criteria in accordance with Kidd et al. [23], requiring that the alignment covered at least 75% of the read length, and that the sequences were at least 95% identical.

We obtained an average of ~ 32.3 million reads per individual with the Expanded Exome Capture Sequencing approach. Of those, 2.67% did not align to the human genome hg19 (~ 690,000 reads per individual) (Supplementary Fig. 1). After quality control, an average of ~ 307,000 high-quality non-human reads per individual were aligned against the microbiome reference genomes of the Human Microbiome Project (HMP) [48] and we built an abundance table with the number of metagenomic reads aligned against each microbial species in each sample. An abundance table was also constructed at the genus level by merging species of the same genus.

### Statistical analyses

All analyses were carried out using R studio version 4.1.1717 [50] at the genus level, unless indicated otherwise. We estimated alpha diversity (diversity within individuals) using the Shannon index [51] with the function “diversity” from the vegan v2.5-7 package [52] after rarefying all samples to a depth of 29,184 reads per sample, corresponding to the minimum number of reads obtained in an individual. Beta diversity (diversity between individuals) was calculated using the Bray–Curtis dissimilarity [53] with the function “vegdist” from the package vegan v2.5-7. Prior to calculating the Bray-Curtis dissimilarity values, we normalized the read counts by applying a variance-stabilizing transformation (VST) using DESeq2 [54] as suggested in McMurdie and Holmes [55]. The VST normalization takes into account that total reads (library size) per sample may differ between samples by orders of magnitude, a fact that should be considered when comparing samples. To evaluate whether differences existed across and between distributions, we used Kruskal-Wallis and Mann-Whitney U tests, respectively, and a Benjamini-Hochberg FDR correction for multiple testing was applied (adjusted p_value < 0.05).

To explore how individuals clustered according to their microbiome profiles, we used a non-metric multidimensional scaling (NMDS) plot and a correspondence analysis (CA) based on genera counts after applying VST. The NMDS was performed on the Bray-Curtis dissimilarity matrix using the function “isoMDS” with default parameters from the package MASS v7.3.54 [56] (Fig. 3A). The CA was performed using the function “dudi.coa” from the package ade4 v1.7-13 [57] and visualized with the function “fviz_ca” from the package factoextra v1.0.7 [58] (Fig. 4).

**Fig. 2:**
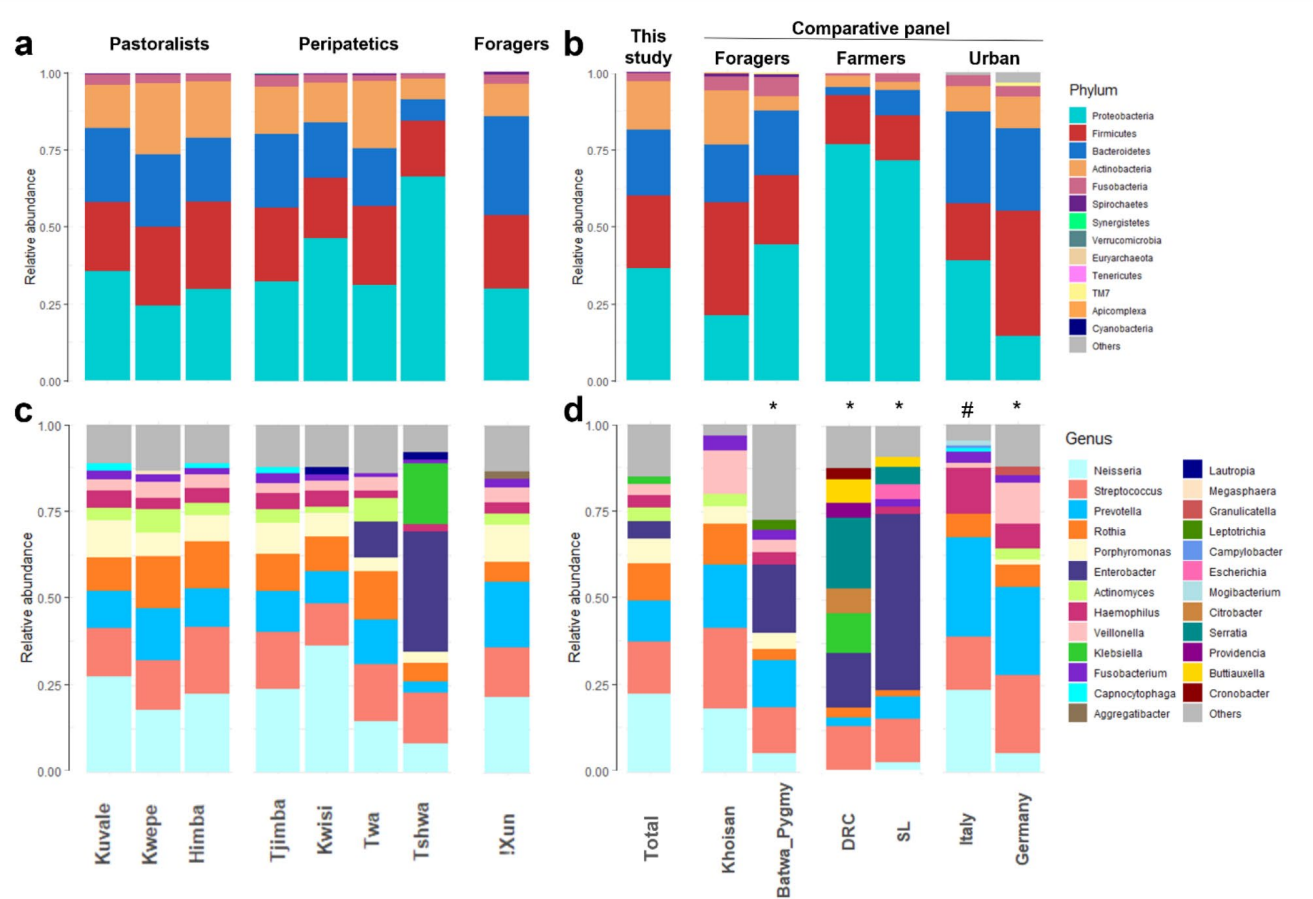
Salivary microbial composition. Relative abundance of phyla **(A, B)** and the ten most frequent genera **(C, D)** in the analyzed southern African populations **(A, C)**, and in a comparative panel of populations from Africa and Europe **(B, D)**. Frequencies for the total population in this study are averages across the 52 individuals. The comparative panel includes data for “Khoisan” foragers from southern Africa also obtained through an Exome capture approach [23]; Batwa Rainforest Hunter-Gatherers (“Pygmies”), Democratic Republic of Congo (DRC), Sierra Leone (SL) [12]; Italy [59]; Germany [16]. Note: * Data from partial 16S rRNA sequences; # Data from whole-genome sequencing

In order to compare the microbiome composition of the sampled populations with those from the literature, we included a panel of salivary microbiome data from four African and two European sample populations. The African population samples include different genetic backgrounds and subsistence strategies: the ǂKhomani foragers from South Africa [23], the Batwa foragers from Uganda, and two agricultural groups from the Democratic Republic of Congo (DRC) and Sierra Leone (SL) [12]. The European samples consist of Italians [59] and Germans [16]. These studies were carried out using different methodologies: while Nasidze et al. [12] and Li et al. [16] used amplicon amplification of hypervariable fragments V1 and V2 of the 16S rRNA gene, Caselli et al. [59] used whole-genome sequencing, and Kidd et al. [23] whole-exome sequencing (WES), corresponding to the approach used in the present study. Since no information on read counts per genus was available for all six populations of the comparative panel, we calculated F_*st*_ values [60] based on the relative frequencies of the ten most frequent genera shown in Fig. 2 with the PHYLIP software [61] and visualized them through an NMDS plot (Supplementary Fig. 5).

Correlations between the relative abundances of the ten most frequent genera have been assessed using the Pearson correlation coefficient. For the Angolan groups, we additionally calculated the correlation between microbiome (Bray-Curtis distances) and genetic data (F_*st*_ distances using mtDNA and Y-chromosome [25, 26]) by means of Mantel tests [62]. Differential abundance (DA) of taxa between all population pairs and between the three subsistence methods (foraging, pastoralist, peripatetic) was calculated using DESeq2, which provides false discovery rate (FDR) adjusted p-values (Supplementary Tables 4–7).

## Results

### Oral microbiome composition

We identified a total of nine phyla in the eight sampled groups from Angola and Zimbabwe: *Proteobacteria*, *Firmicutes*, *Bacteroidetes*, *Actinobacteria*, *Fusobacteria*, *Spirochaetes*, *Synergistetes*, *Verrucomicrobia*, and *Euryarchaeota* (Supplementary Table 1), with four phyla (*Proteobacteria*, *Firmicutes*, *Bacteroidetes*, *Actinobacteria*) recruiting 96% of the reads (Fig. 2A). These four phyla are also predominant (92–99%) in a comparative panel of four African and two European populations (Fig. 2B).

The nine phyla could be additionally broken down into 206 genera (Supplementary Table 2) and 574 taxa at higher resolution, including 468 identified species (Supplementary Table 3). *Neisseria* (phylum *Proteobacteria*), *Streptococcus* (*Firmicutes*), *Prevotella* and *Porphyromonas* (both *Bacteroidetes*), and *Rothia* (*Actinobacteria*) represent between 62% and 74% of the microbiome communities of Angolan populations (Fig. 2C). These well-known genera of the oral microbiome are also abundant in other African and European populations from the comparative panel (Fig. 2D).

As it has been suggested that different genera may associate to form distinct communities with particular microbial combinations [63, 64], we have assessed patterns of co-occurrence by calculating Pearson correlations between the relative abundances of the 10 most frequent genera that were found in the 52 sampled individuals. We found four significant positive correlations after FDR correction (Supplementary Fig. 3A-D): *Prevotella* with *Veillonella* (r = 0.74; p < 0.001); *Actinomyces* with *Veillonella* (r = 0.58; p < 0.001); *Prevotella* with *Actinomyces* (r = 0.58; p < 0.001); and *Neisseria* with *Haemophilus* (r = 0.54; p < 0.001). In contrast, the frequencies of *Neisseria* were negatively correlated with *Actinomyces* (r = -0.43; p = 0.01), *Prevotella* (r = -0.40; p = 0.03) and *Veillonella* (r = -0.37; p = 0.04) (Supplementary Fig. 3E-G), thus clearly revealing two alternative microbial combinations: *Prevotella-Veillonella-Actinomyces* and *Neisseria-Haemophilus*.

When microbiome profiles are compared across the studied populations, the Tshwa from Zimbabwe stand out for their unusually high frequency of the *Proteobacteria* phylum (66% in the Tshwa vs. 24–46% in the seven Angolan populations), which is also common in the Democratic Republic of Congo (DRC) (77%) and Sierra Leone (SL) (72%) [12] (Fig. 2A and B). These elevated frequencies are mostly due to the *Enterobacter* and *Klebsiella* genera, which represent 52% of all reads in the Tshwa, 51% in SL and 27% in the DRC (Fig. 2C and D). A high frequency of *Enterobacter* and *Klebsiella* (22%) was also found among the Batwa from Uganda [12] (Fig. 2D).

Consideration of individual relative abundance profiles reveals that the Tshwa have very uneven genera distributions, with three out of five individuals displaying microbiomes that are dominated by two genera: *Klebsiella* in individual ZIM28 (86% of 2.45 million reads) and *Enterobacter* in individuals ZIM32 (84% of 361,940 reads) and ZIM39 (86% of 790,392 reads) (Supplementary Fig. 2G and Supplementary Table 2). A high frequency (72% of 973,242 reads) of the *Enterobacter* genus was also found in a single Twa individual (AngH229) from Angola (Supplementary Fig. 2F).

When compared with the other populations analyzed here, the unusual character of the microbiome profile of the Tshwa is further reflected in the low alpha diversity calculated after rarefying the number of reads, which measures the variability of the microbial compositions of each sampled individual (Supplementary Fig. 4A). However, no significant differences existed in alpha values between populations grouped according to subsistence patterns (Supplementary Fig. 4B), nor between sexes.

### Clustering analysis based on microbial profiles

**Fig. 3:**
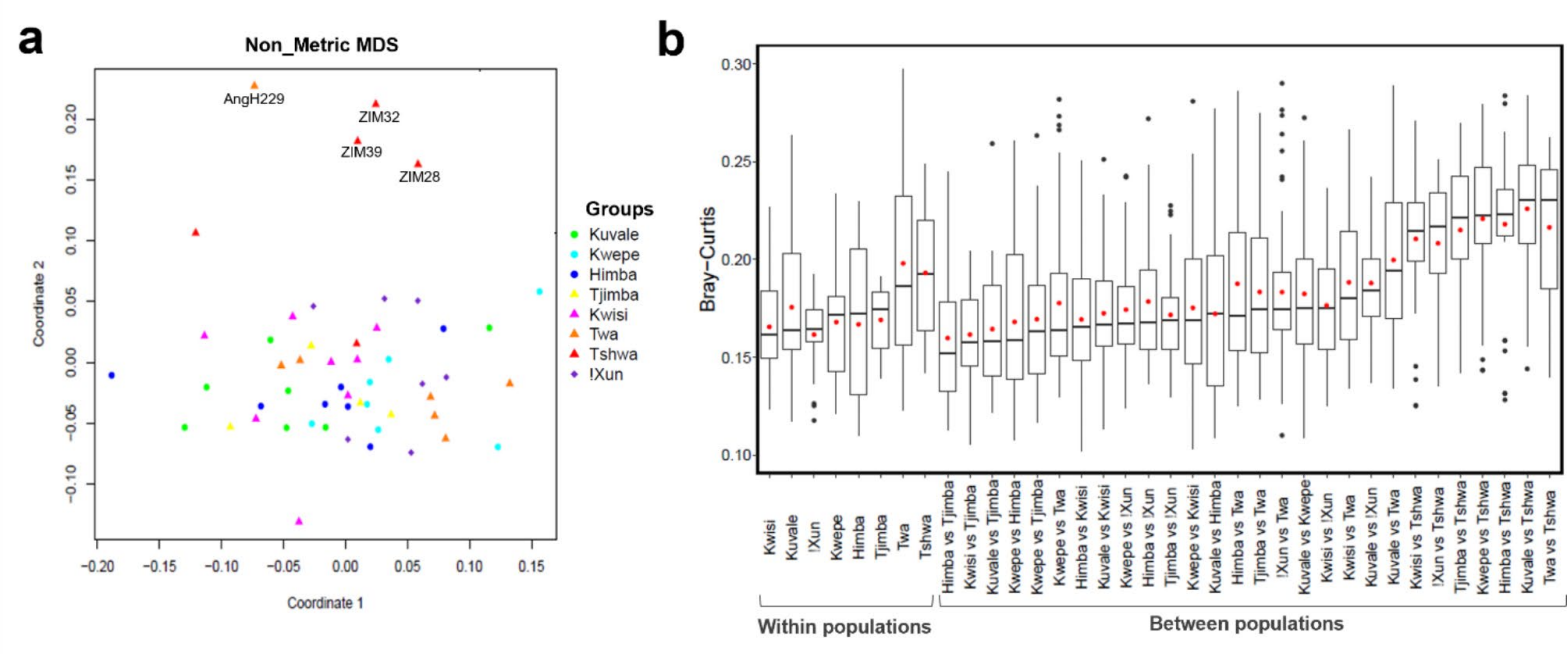
Pairwise Bray-Curtis dissimilarity values. **(A)** Non-metric MDS depicting inter-individual Bray-Curtis dissimilarity values. Colored symbols represent individuals from different populations. Circles, triangles, and diamond symbols represent pastoralists, peripatetics, and foragers, respectively. Tshwa (ZIM28, ZIM32, ZIM39) and Twa (AngH229) individuals with one *Enterobacteriaceae* taxon at a frequency > 70% are indicated. **(B)** Distribution of mean pairwise Bray-Curtis values within and between populations. Horizontal lines inside boxplots represent the median, and red circles correspond to mean values

We carried out clustering analyses in order to investigate if individual differences in the composition of bacteria genera are structured by ethnic group, subsistence pattern, or geography. Figure 3A shows a non-metric multidimensional scaling (NMDS) plot based on pairwise Bray-Curtis dissimilarity values between individual microbiome profiles, calculated after a Variance Stabilization Transformation (VST) to correct for unequal library sizes [55]. Apart from the clear differentiation of the Tshwa and Twa individuals with unique microbiome profiles (ZIM28, ZIM32, ZIM39 and AngH229), most individuals are scattered across the plot without any clear clustering (Fig. 3A). A similar result was obtained when comparisons were done at the species level (not shown).

In agreement with the NMDS plot, the distributions of Bray-Curtis distances show that, except for comparisons involving the Tshwa, microbiome differences between individuals from the same population are similar to those between individuals from different populations (Fig. 3B).

The same pattern was observed when the distributions of Bray-Curtis distances were calculated within and between subsistence patterns or sexes (not shown).

This general lack of structuring is also reflected in the absence of correlation between average Bray-Curtis distances in microbiome composition across populations and F_*st*_ genetic distances calculated with available mtDNA (Mantel test r = 11, p = 0.33) and Y-chromosome data (r=-0.13, p = 0.63) from Angola [25, 26]. In addition, the differences in microbiome composition within Angola are not correlated with geographic distances among populations (r = 0.06; p = 0.25). Only when the outlying Tshwa from Zimbabwe are included in the comparisons can a significant correlation with geographic distance (r = 0.39; p = 0.002) be observed, suggesting that there is no robust association between microbiome differentiation and geographic distance in our data.

**Fig. 4:**
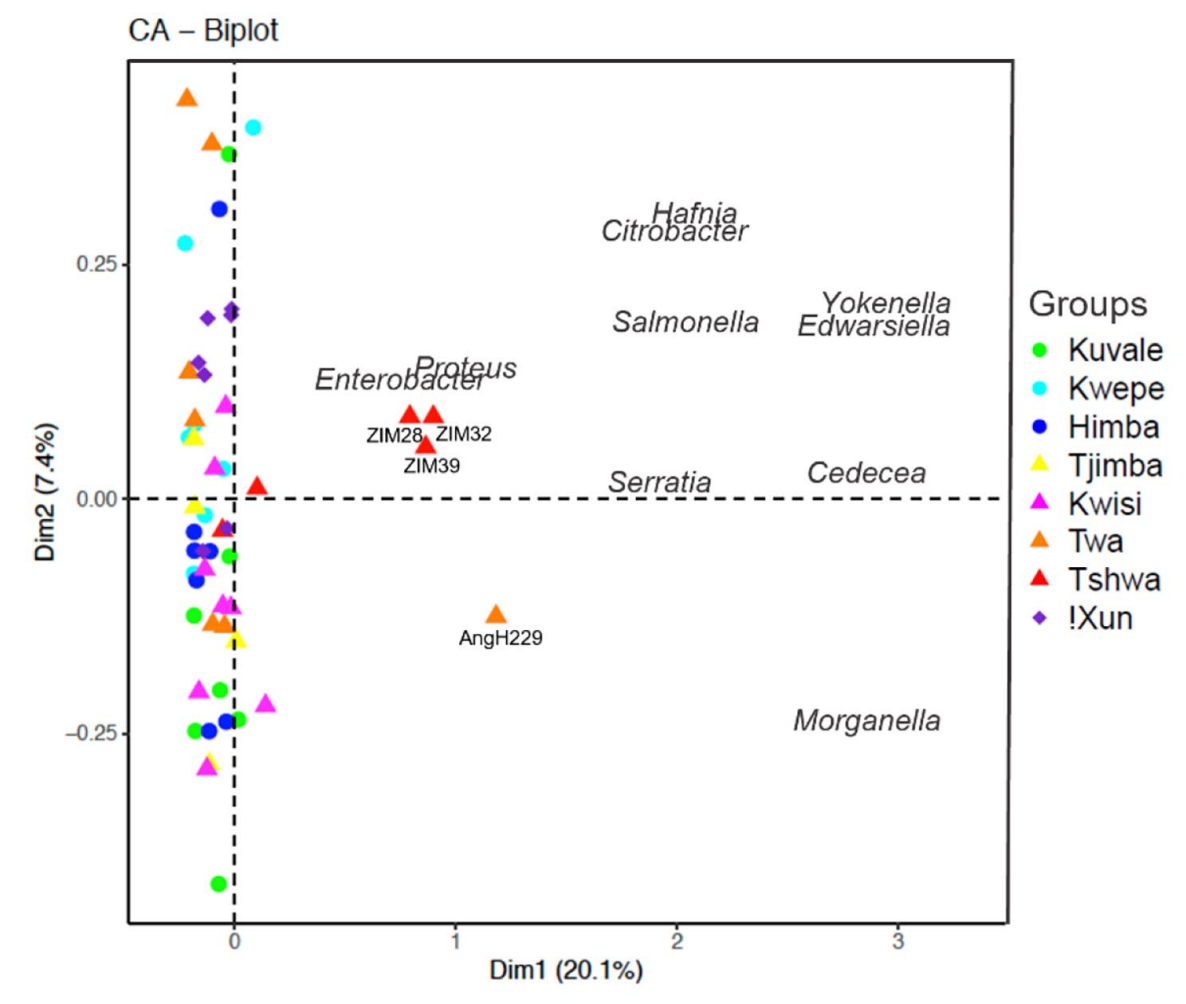
Correspondence analysis (CA). CA based on genera counts after VST normalizing. Colored symbols represent individuals belonging to different populations. Circles, triangles, and diamond symbols represent pastoralists, peripatetics, and foragers, respectively. The ten genera with the highest contribution are shown in the plot. Tshwa (ZIM28, ZIM32; ZIM39) and Twa (AngH229) individuals with outstanding microbiome profiles are also indicated

In order to further identify the most important genera driving microbiome differentiation we additionally performed a correspondence analysis (CA) based on genera counts with a VST. The resulting CA plot is consistent with the patterns observed in the NMDS plot and shows that the 10 genera most strongly separating the samples are *Cedecea*, *Citrobacter*, *Edwarsiella*, *Enterobacter*, *Hafnia*, *Morganella*, *Proteus*, *Salmonella*, *Serratia*, and *Yokenella*, all belonging to the *Proteobacteria* phylum (Fig. 4).

We have also attempted to compare the microbiome profiles of our sampled groups with published data on other African and European populations from the comparative panel shown in Fig. 2. As read counts could not be obtained for all published groups, and genera abundances were available only for the most frequent genera, we carried out an NMDS analysis based on F_*st*_-like distances between populations, considering the relative frequencies of their 10 most common genera (Supplementary Fig. 5). In accordance with the profiles displayed in Fig. 2, the observed patterns of microbiome differentiation show that the Tshwa from Zimbabwe, the Batwa from Uganda, and the individuals from DRC and SL – all with high frequencies of *Enterobacteriaceae* – appear as outliers. All other populations, including the various Angolan groups, Germans, Italians and the “Khoisan” foragers from South Africa, have similar microbiome compositions and do not form any apparent clusters (Supplementary Fig. 5).

## Differential abundance of taxa across populations

**Fig. 5:**
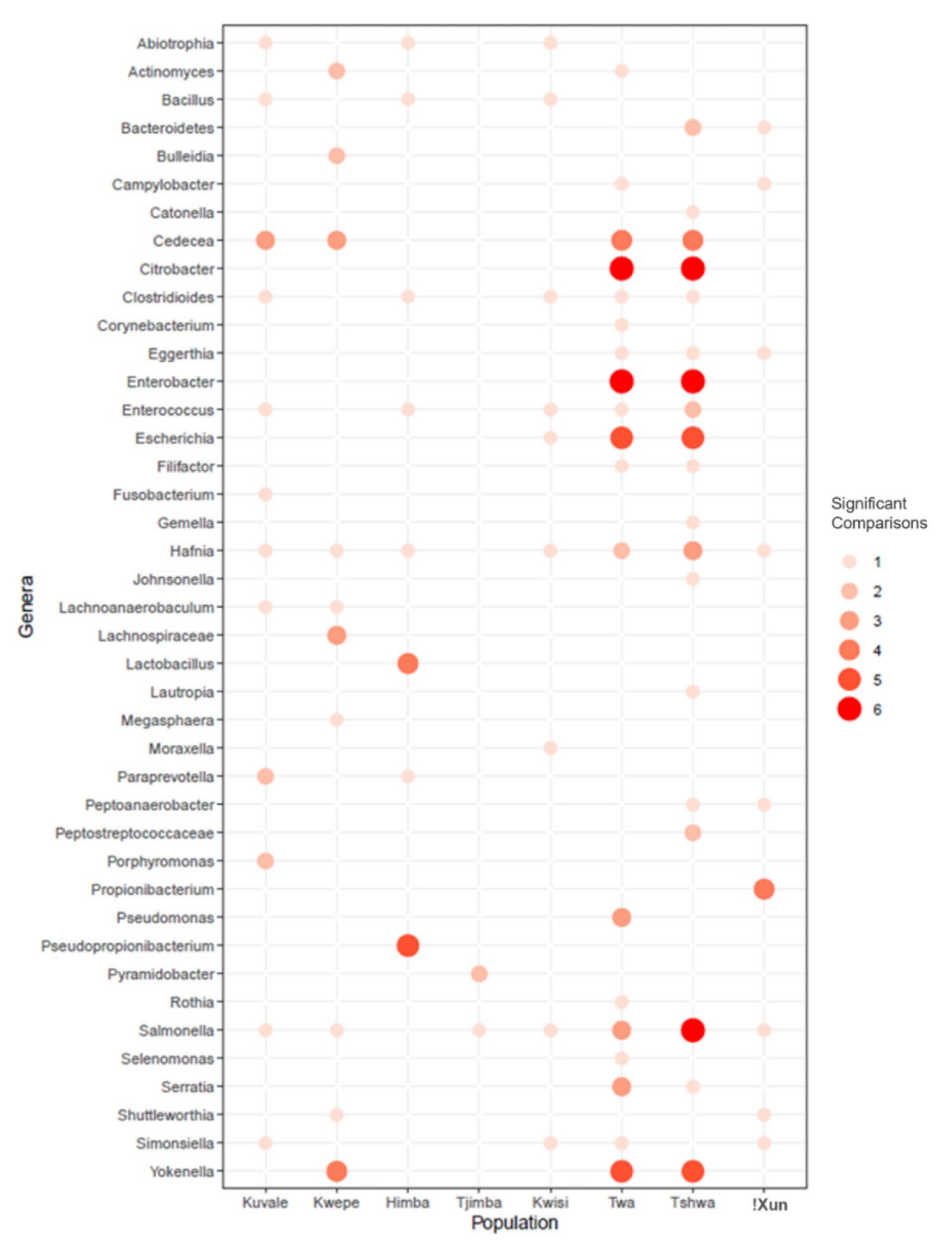
Differential Abundance (DA) analysis between populations at the genus level. Dot plot showing the number of pairwise comparisons in which a genus (Y axis) was overrepresented in a given population (X axis)

To investigate whether specific taxa vary significantly in frequency among populations, despite the general absence of structuring in the salivary microbiome composition, we used DESeq2 to assess the differential abundance (DA) of the 206 identified genera between pairs of populations, in a total of 5768 pairwise comparisons. After correcting for multiple testing, we identified 171 significant comparisons involving 41 genera (Supplementary Table 4). Thirteen out of the 41 genera were found to be overrepresented in at least three pairwise comparisons involving a specific population (Fig. 5). Seven out of these 13 genera are among the most important genera driving microbiome differentiation in the CA plot (Fig. 4) and are overrepresented in the Tshwa and/or the Twa: *Cedecea*, *Citrobacter*, *Enterobacter*, *Salmonella*, *Serratia*, *Yokenella* (all from the *Enterobacteriaceae* family), and *Hafnia* (from the *Hafniaceae* family) (Fig. 5). Of note are also the overrepresentations of the fermentation-associated *Lactobacillus* and *Pseudopropionibacterium* genera in the pastoralist Himba, who are known to consume large amounts of fermented milk [65–69] (Fig. 5).

We further extended the DA analysis to taxa identified at the species level and found that several *Enterobacteriaceae* species that are enriched in the Tshwa and/or Twa are known opportunistic pathogens involved in health-care infections and/or immunocompromised patients: *Cedecea davisae*, *Escherichia coli*, *Yokenella regensburguei*, several species of *Citrobacter* (*C. freundii*, *C. youngae*, *C. koseri*) and *Klebsiella* (*K. oxytoca*, *K. pneumoniae*) [70] (Supplementary Table 5 and Supplementary Fig. 6).

To identify taxa that are enriched in groups with a particular subsistence pattern, we performed a DA analysis comparing genera and species levels between foragers, pastoralists and peripatetics (Supplementary Tables 6 and 7, Supplementary Fig. 7). In accordance with the DA analysis carried out between populations, most significant differential abundances were shown by taxa enriched in the peripatetic groups (Supplementary Fig. 7). Nonetheless, grouping individuals according to their livelihood allowed us to increase the statistical power and detect interesting taxa, like *Bifidobacteria breve* that is more abundant in foragers than in both pastoralists and peripatetics (Supplementary Fig. 7). *B. breve* is a probiotic species with important benefits, which is used to prevent intestinal inflammation, as well as in the treatment of diarrhea and constipation [71].

## Discussion

To obtain a more accurate picture of the human oral microbiome composition and its role in human health and disease, comparative data from a broad range of populations from different geographical areas following diverse subsistence strategies is needed. Here, we have analyzed the salivary microbiome profiles of eight diverse ethnolinguistic groups from Angola and Zimbabwe who explore different ecological settings and livelihoods, including pastoralism, foraging, and peripatetic lifeways.

We found similar amounts of inter-individual microbiome differentiation within and between groups, resulting in a lack of population structure based on the oral microbiome composition, in agreement with what was observed by Nasidze et al. [11] for other groups. This pattern suggests that the diversity of bacterial communities in the studied groups appears to be more influenced by individual factors than by genetic differentiation between populations.

However, since our study is based on low sample sizes, encompassing small, inbred groups, a broader analysis of more individuals would be needed to confirm these results. In addition, our data was collected in the context of population history research and therefore lacks individual metadata on diet, hygiene habits and general health status, which may shed further light on the observed patterns of variation. Notwithstanding these limitations, our results revealed taxa compositions and distribution patterns consistent with those observed in other studies based on different methodologies [11, 12, 16, 23, 59, 64, 72, 73]. This similarity further indicates that our data did not present a batch effect, suggesting that non-human reads obtained from Exome Capture Sequencing on saliva samples allow for a faithful characterization of the saliva microbiome [23].

In addition to general patterns of diversity observed in all sampled groups, we found that four individuals, three Tshwa from Zimbabwe and one Twa from Angola, display extremely differentiated microbiome profiles. This differentiation is due to a high proportion of pathogenic taxa, especially from the *Enterobacteriaceae* family (e.g., *E. hormaechei*, *E. cancerogenus*, and *Klebsiella michiganensis*), which are linked to poor sanitary conditions as well as nosocomial infections affecting immunocompromised patients [70]. Previous studies focusing on the role of HIV in shaping the salivary microbiome have shown that a compromised immune system is vulnerable to microbial changes, leading to elevated frequencies of *Enterobacteriaceae* among HIV-positive individuals [74–78]. While we do not have data on the health status and sanitary conditions of the sampled individuals, our observations in the field align with previous studies which suggest that especially the Tshwa experience considerable levels of social marginalization and poverty, including lack of access to clean water supplies and regular alimentation [42]. Furthermore, data released by the World Health Organization (WHO) suggests that in 2018, Zimbabwe presented the 4th highest HIV prevalence from a total of 36 African countries (https://www.afro.who.int/health-topics/hivaids), in line with the observation that Tshwa communities are especially affected by HIV [42]. It therefore seems likely that the outstanding microbiome profiles seen in our data could be caused by compromised immunity and a poor nutritional level. Nevertheless, further studies should formally test this.

## Conclusions

Our results provide new insights into the diversity of the salivary microbiome displayed by African populations, focusing on a diverse set of ethnic groups from Angola and Zimbabwe. Rather than aligning with genetic distance, ethnic affiliation or subsistence pattern, inter-individual diversity appears to be related to socio-economic conditions, access to sanitation, and health status. Our findings therefore underline the important role played by the oral microbiome in the context of systemic health.

### Electronic supplementary material

Below is the link to the electronic supplementary material.

Supplementary Material 1

Supplementary Material 2

## Data Availability

The sequence datasets generated for this study can be found in the European Nucleotide Archive (ENA) repository under the PRJEB53437 accession number (https://www.ebi.ac.uk/ena/browser/view/PRJEB53437).
