# Supplementary material for: Oral microbiome homogeneity across diverse human groups from southern Africa: first results from southwestern Angola and Zimbabwe
Source: BMC Microbiol. 2023 Aug 18;23:226. doi: 10.1186/s12866-023-02970-2 (PMC10436416; doi:10.1186/s12866-023-02970-2)
Supplement: Supplementary file 2 — Supplementary Material 2 [file 12866_2023_2970_MOESM2_ESM.pdf]

Supplementary Material

Supplementary Figures

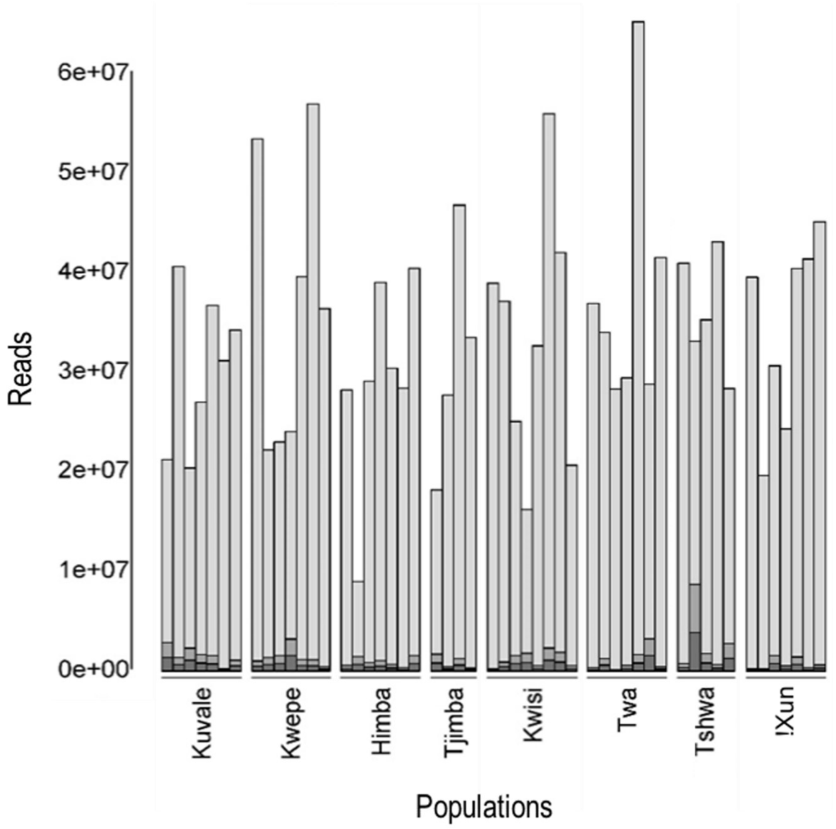

Supplementary Fig. 1 Number of reads per individual at different stages of the bioinformatic pipeline. Light grey bars represent the total number of reads obtained for each sample using Expanded Exome capture sequencing. Grey bars represent the number of unmapped reads after the alignment with the human genome and dark grey bars represent the number of high-quality unmapped reads after filtering with Prinseq tool.

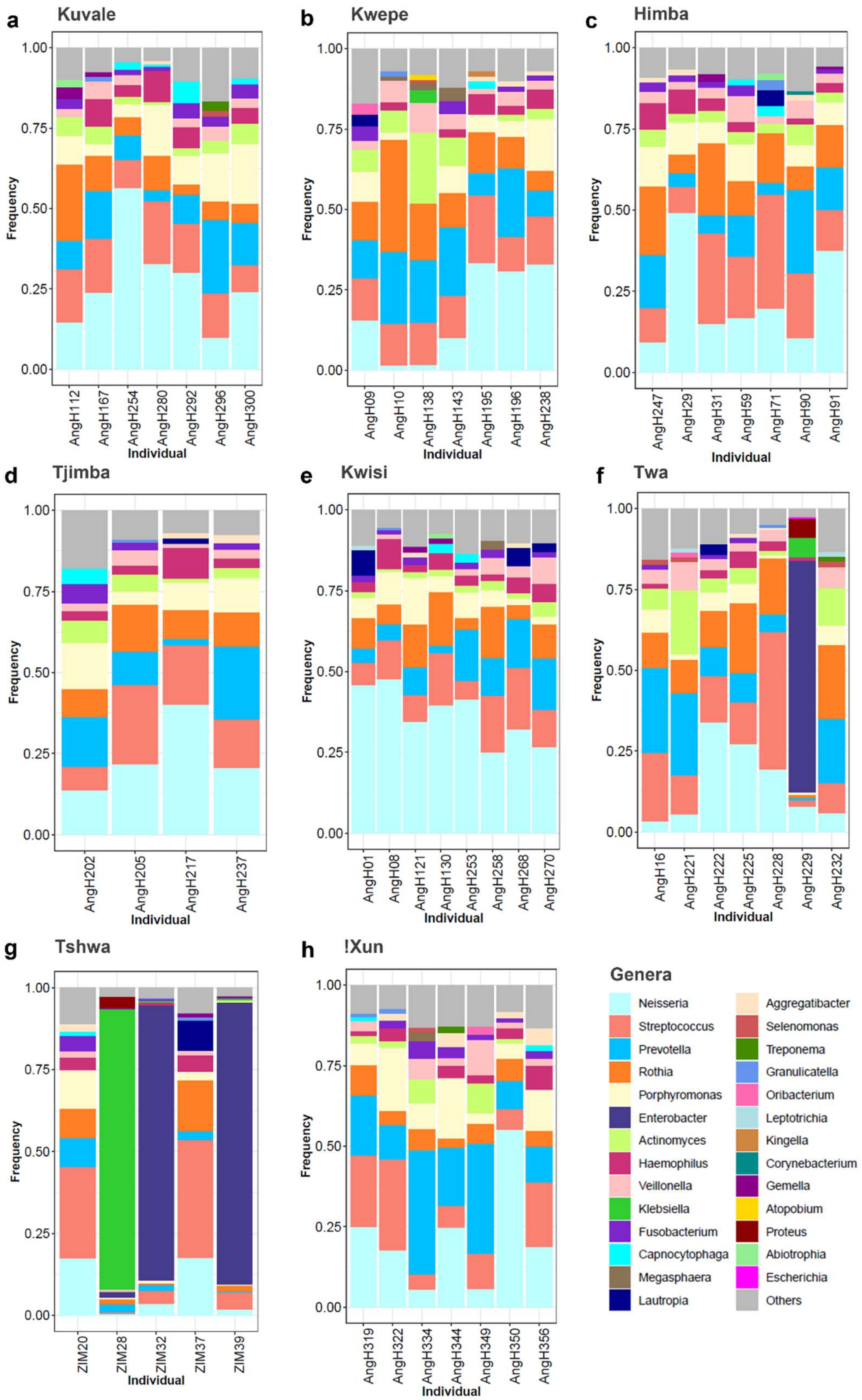

Supplementary Fig. 2 Relative abundance of the ten most frequent genera found in each sampled individual. (A-C) Pastoralists; (D-G) Peripatetics; (H) Foragers.

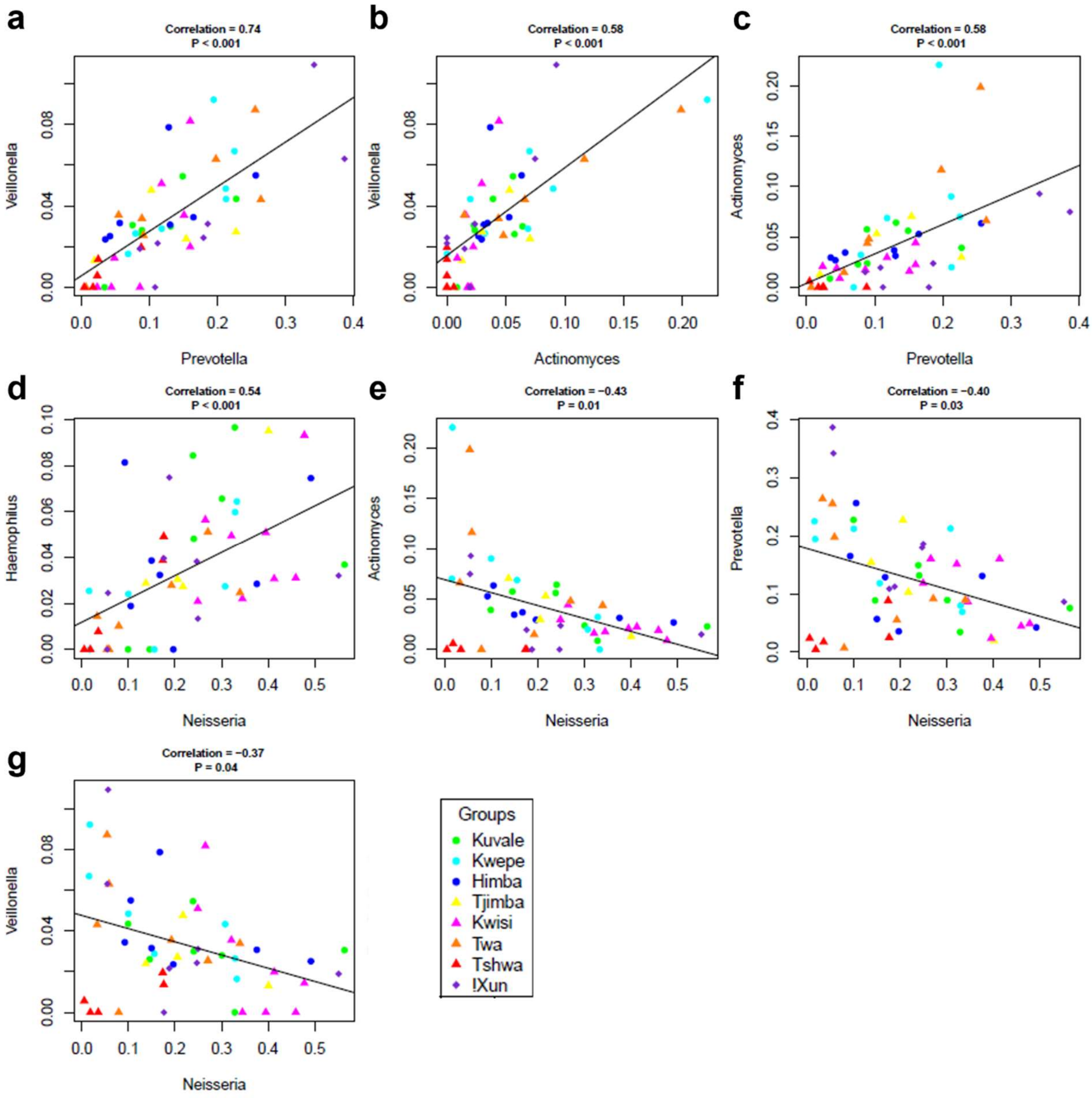

Supplementary Fig. 3 Plots showing the relative abundance of genera with significant correlations after FDR. (A-D) Positive correlations; (E-G) Negative correlations.

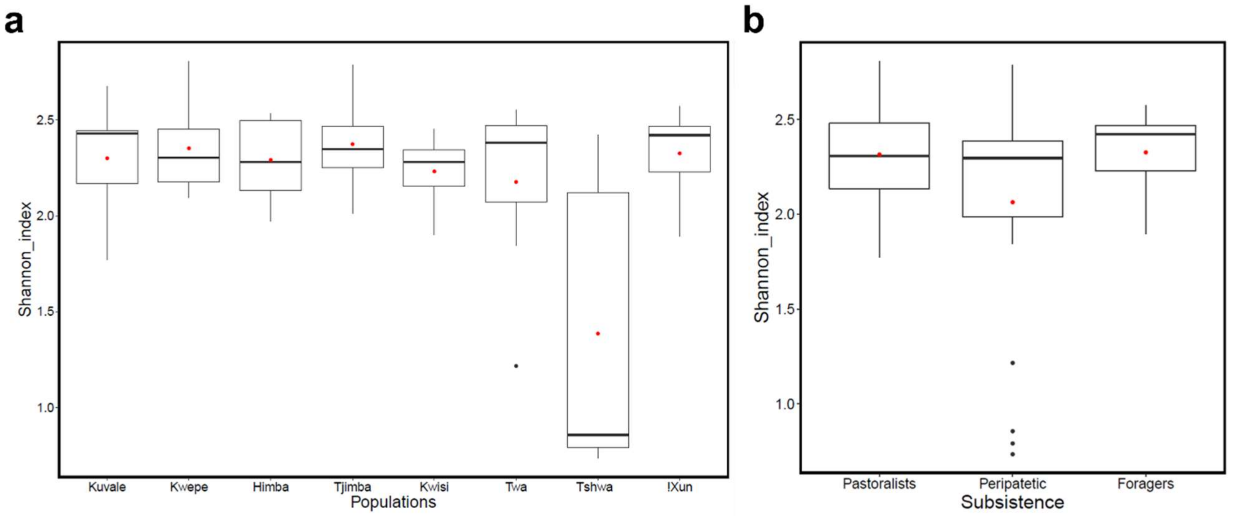

Supplementary Fig. 4 Distribution of alpha diversity (Shannon index) values at the genera level calculated for all individuals grouped by (A) population, and (B) subsistence pattern. Horizontal lines inside boxplots represent the median and red circles correspond to the population mean values (Kuvale: 2.30, Kwepe: 2.35, Himba: 2.29, Tjimba: 2.37, Kwisi: 2.23, Twa: 2.18, Tshwa: 1.39, !Xun: 2.33, Pastoralists: 2.32, Peripatetics: 2.06, Foragers: 2.33).

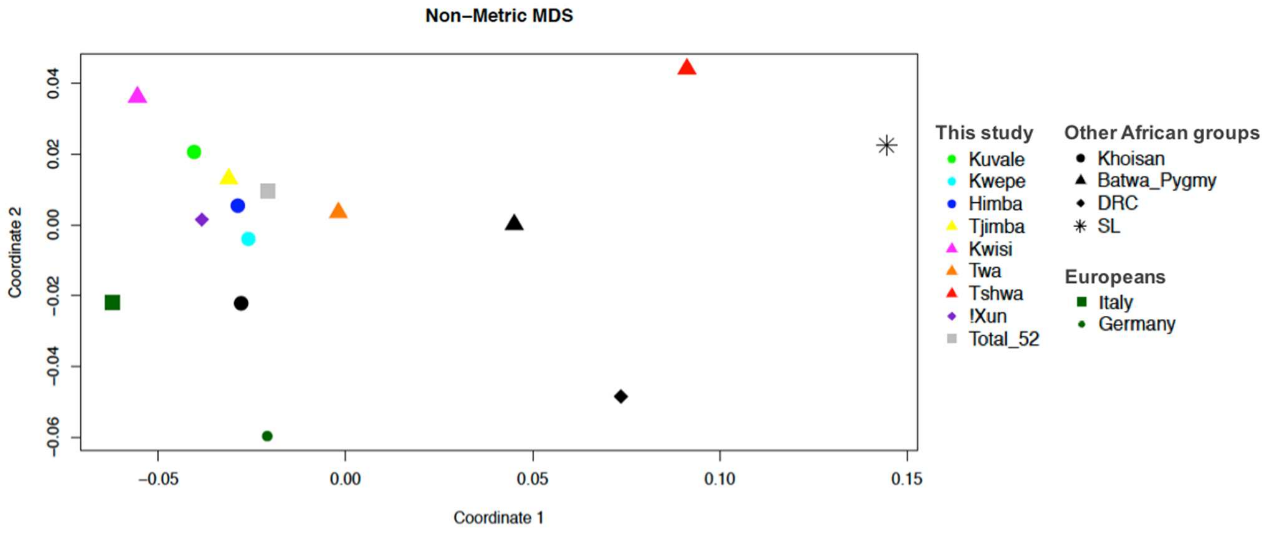

Supplementary Fig. 5 Non-metric MDS depicting the F_{st}-like values between populations based on the relative frequencies of the most common genera as shown in Fig. 2. Colored symbols represent populations. The group named “Total_52” refers to the general population of this work, which includes 52 individuals.

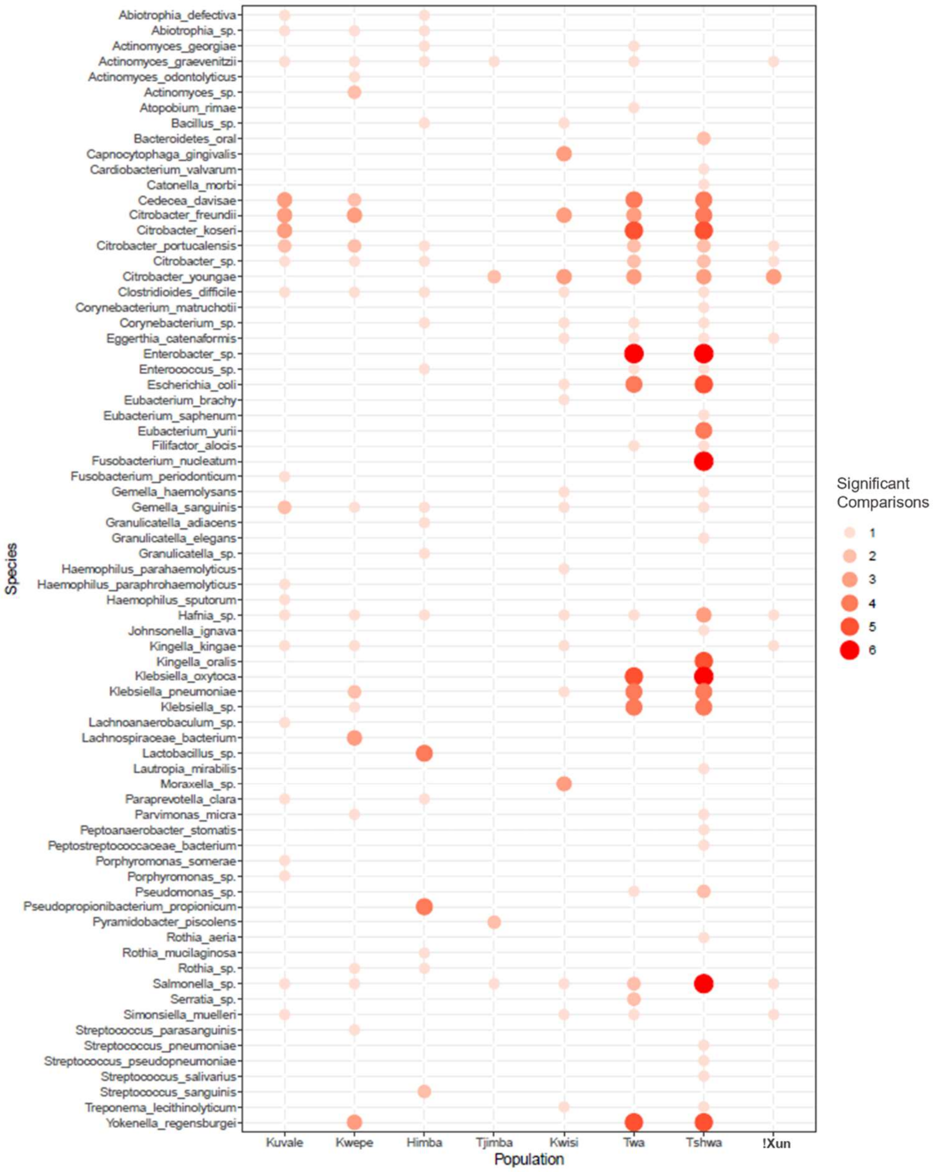

Supplementary Fig. 6 Differential Abundance (DA) analysis between populations at the species level. Dot plot showing the number of pairwise comparisons in which a taxon (Y axis) was overrepresented in a given population (X axis).

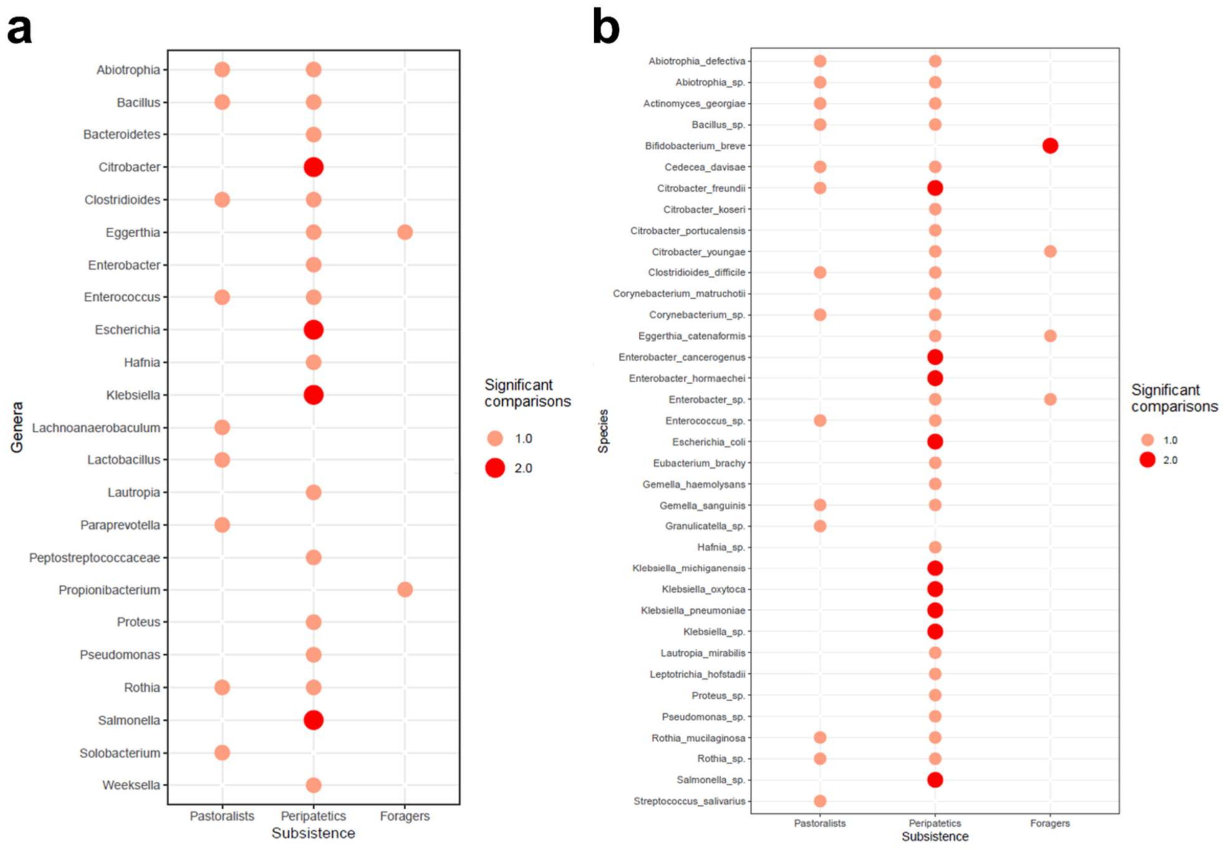

Supplementary Fig. 7 Differential Abundance (DA) analysis between subsistence patterns at the genera (A) and species (B) level. Dot plot showing the number of pairwise comparisons in which a taxon (Y axis) was overrepresented in a given subsistence category (X axis).
